# Decorticating and Grafting Technique in Posterior Instrumented Spinal Fusion for Idiopathic Scoliosis Correction Surgery

**DOI:** 10.7759/cureus.88313

**Published:** 2025-07-19

**Authors:** Stavros Goumenos, Dimitrios V Papadopoulos, Paris Christogeorgos, Ioannis Trikoupis, Spyridon Sioutis, Anastasios G Roustemis, Ioannis Chatzikomninos, Anastasios Kalampokis, Vasilios Igoumenou, Peter R Loughenbury, Almas Khan, Konstantinos Soultanis, Panayiotis J Papagelopoulos

**Affiliations:** 1 Center for Musculoskeletal Surgery (CMSC), Charité - Universitätsmedizin Berlin, Berlin, DEU; 2 Second Department of Orthopaedic Surgery, National and Kapodistrian University of Athens, School of Medicine, Konstantopouleio General Hospital, Athens, GRC; 3 Department of Spine Surgery, Metropolitan General Hospital, Athens, GRC; 4 First Department of Orthopaedic Surgery, National and Kapodistrian University of Athens, School of Medicine, Attikon University General Hospital, Athens, GRC; 5 First Department of Orthopaedic Surgery, National and Kapodistrian University of Athens, School of Medicine, Athens, GRC; 6 Department of Orthopaedic Surgery and Traumatology, Attikon University General Hospital, Elefsina, GRC; 7 Department of Spine Surgery and Scoliosis, KAT General Hospital, Athens, GRC; 8 Department of Orthopaedic Surgery, Eifelklinik St. Brigida GmbH and Co. KG, Simmerath, DEU; 9 Department of Spine Surgery, Leeds Teaching Hospitals NHS Trust, Leeds, GBR

**Keywords:** idiopathic scoliosis, posterior decortication, spinal fusion, spinal grafting, spine

## Abstract

The success rate of spinal deformity correction surgeries relies on a robust long-term vertebral arthrodesis. However, the non-union rate in spinal fusion for lumbar or thoracic deformities is still high, despite the recent advances in implant design, surgical techniques, and bone grafting technology. Meticulous and thorough decortication is of great importance, while bone grafting is considered to enhance the fusion rate, improving the results of these procedures. The aim of this technical note is to thoroughly describe in a stepwise fashion a surgical technique for complete midline and lateral posterior decortication and bone grafting in posterior instrumented spinal fusion surgery for scoliosis deformity correction surgery. Critical steps of decortication include obliteration of the facet joints and decortication of the surfaces of the pars interarticularis, lamina, later aspects of the articulating facets, and transverse processes (TPs), as well as removal of all the excessive surrounding soft tissues. For bone grafting, the spinous processes of the vertebrae are hemi-sectioned at the base, divided in half, while a novel ceramic bio-glass is also used as a graft expander. Moreover, recent literature regarding posterior elements decortication and bone grafting in posterior instrumented spinal fusion is presented.

## Introduction

Posterior instrumented spinal fusion is one of the most common spinal procedures, indicated for various pathologies such as spinal deformities, vertebral fractures, degenerative disk disease, and spondylolisthesis [1]. Since a successful long-term arthrodesis is the paramount goal of an instrumented posterior spinal fusion, there has been much debate regarding the beneficial effect of an extensive posterior elements’ decortication, as well as regarding the optimal implant selection and the adjuvant use of bone grafts [2,3]. Nevertheless, despite all the recent advances in implant design, surgical techniques, and bone graft properties, non-union rates following instrumented posterior spinal fusion are reported to be as high as 35% [4].

Historically, the fusion rate with the use of iliac crest bone graft (ICBG) in posterolateral spinal fusion has been reported to be 50%-90%. Hibbs and Albee were the first to use bone chips from the spinous processes, transverse processes (TPs), laminae, and tibia to enhance fusion, while the concept of decortication of the recipient osseous bed to promote spinal fusion was first introduced by Hibbs in 1911 [5]. The ideal bone graft for spinal fusion should provide osteogenic cells with the potential to differentiate into osteoblasts, osteoinductive factors, and an osteoconductive matrix amenable to the ingrowth of blood vessels and osteoprogenitor cells required for bone formation. Finally, bone grafts should provide adequate structural integrity for mechanical support to the spinal fusion [6]. Although autografts are considered the gold standard in spinal surgeries, they are associated with certain complications, such as seroma/hematoma formation, chronic pain at the donor site, infection, and reduced sensation at the donor site, with an overall donor site morbidity of up to 50% [7-10]. Therefore, there is a great interest in the development of bone graft substitutes such as ceramics and bone morphogenetic proteins that can promote spinal fusion without the additional risk of donor site morbidity. However, to date, there is no graft substitute that has shown superiority in comparison to autologous bone graft. Thus, autograft is still considered the gold standard grafting material for spinal fusion [11-15].

The purpose of this technical note is to document in a stepwise fashion our preferred surgical technique for complete posterior decortication and grafting in idiopathic scoliosis correction surgery, as well as to consolidate and present the current body of evidence regarding posterior element decortication and bone grafting in posterior instrumented spinal fusion.

## Technical report

Under general anesthesia, patients are placed in a prone position on an Allen table. Following thorough skin disinfection and fluoroscopic spinal level check, a midline longitudinal incision is performed. The subcutaneous tissue and deep fascia are incised, and the spinous processes of the selected spinal levels are exposed. Meticulous subperiosteal dissection of the paraspinal muscles is performed using electrocautery to minimize intraoperative bleeding.

**Figure 1:**
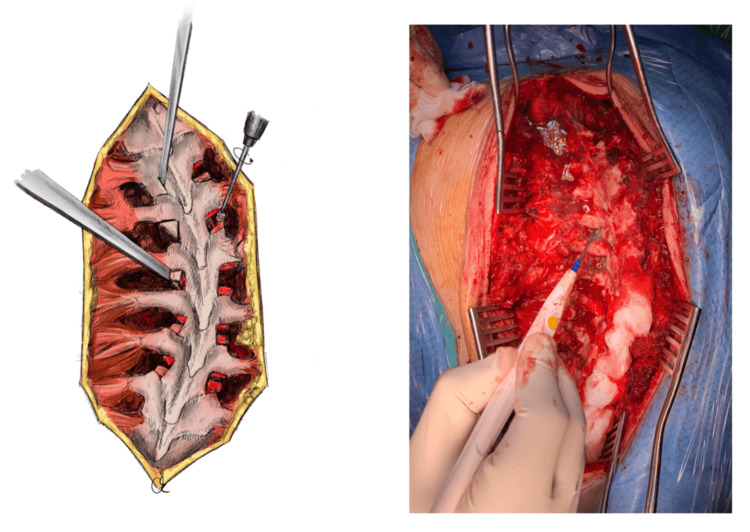
Vertebral facet joint resection and decortication The inferior articular processes of all thoracic vertebrae involved in the posterior fusion are excised using an osteotome, by making two vertical cuts perpendicular to each other, as shown in the illustration (left). The excised osseous parts are collected to be used as part of the autologous bone graft later. The cartilage of the underlying superior articular processes of each facet joint is removed using a burr, as shown in the illustration (left), or diathermy, as shown in the intraoperative picture (right). Illustration by Kimberly Ohm

All facet joints of the selected vertebrae are completely exposed from their capsules, and all remaining soft tissues are completely debrided. In the thoracic spine, the inferior articular processes of all thoracic vertebrae involved in the posterior fusion are excised using an osteotome or an ultrasonic bone knife, while the cartilage of the underlying superior articular processes of each facet joint is removed using a burr or diathermy to facilitate fusion. Regarding the lumbar spine, the facet joints of all lumbar levels involved in the posterior fusion are excised using an osteotome or a rongeur (Figure 1).

**Figure 2:**
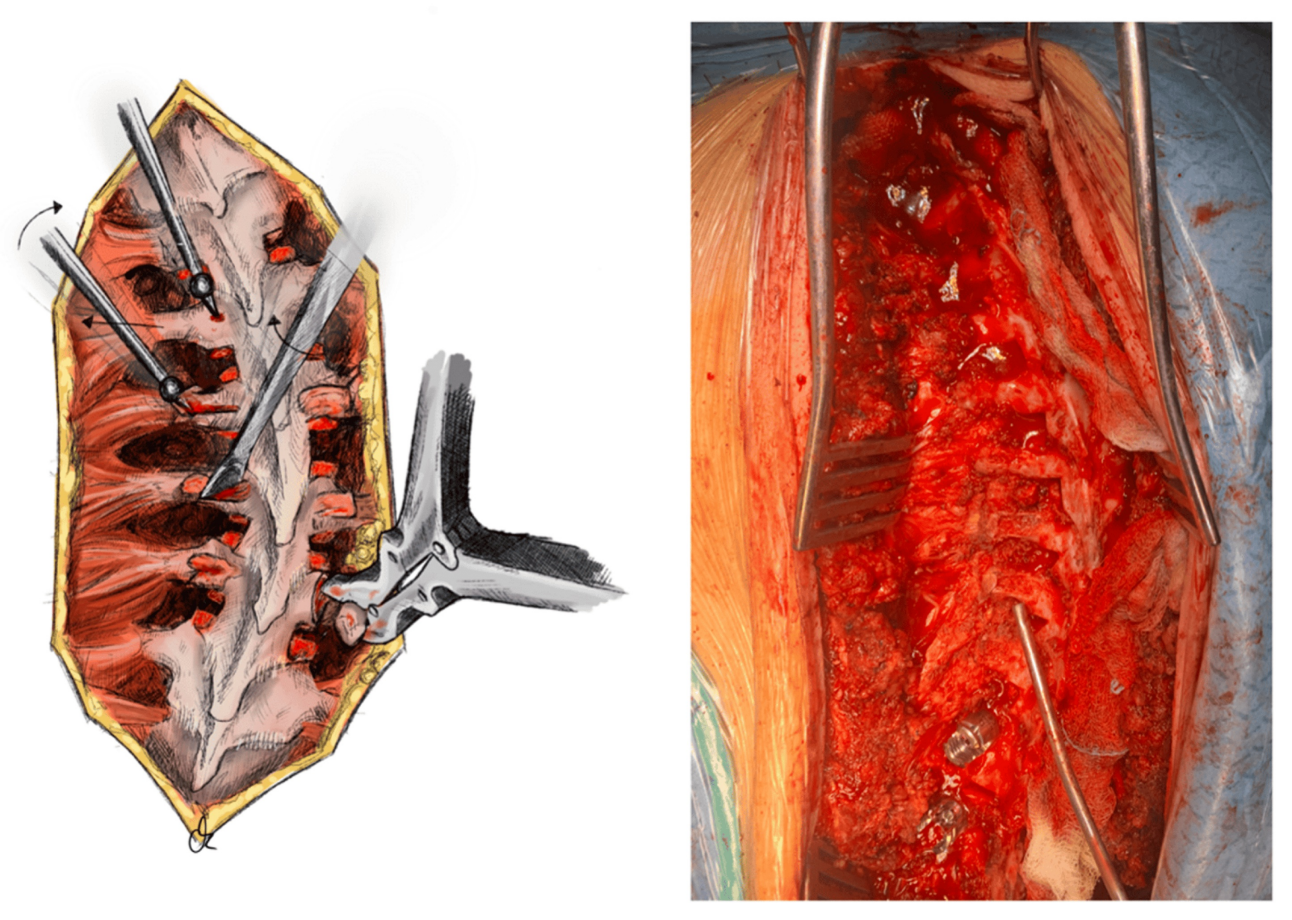
Pars interarticularis and transverse process decortication Using the lateral border of the pars and the respective TP as landmarks, the entry point for each pedicle screw is prepared with an awl. Prior to pedicle screw insertion, the awl is placed in the already prepared hole, and being levered like a pickaxe, it decorticates the respective transverse process by creating a small fine trench across the inferior or superior margin of its posterior cortex, in a medial to lateral direction. Right after, a small Cobb periosteal elevator or a fine osteotome is placed in the prepared trench and is used as a counter-lever to raise and unfold the posterior aspect of the TP in a craniocaudal direction, with the intact anterior cortex serving as a hinge, as demonstrated from cranial to caudal direction on the left side of the illustration above (left). Alternatively, a rongeur can also be used to decorticate the dorsal aspect of the transverse process, as shown on the right side of the illustration above (left) and as depicted and indicated by the suction tip in the intraoperative picture (right). TP: transverse process Illustration by Kimberly Ohm

Using the lateral border of the pars and the respective transverse process (TP) as landmarks, the entry point for each pedicle screw is identified and initially marked with the use of an awl [16]. Prior to pedicle screw insertion, the awl is placed in the already prepared hole, and being levered like a pickaxe, it decorticates the respective transverse process by creating a small fine trench across the inferior or superior margin of its posterior cortex, in a medial-to-lateral direction. As the second step of TP decortication, especially in case of wide TPs, a small Cobb periosteal elevator or a fine osteotome is placed in the prepared trench and is used as a counter-lever to raise and unfold the posterior aspect of the TP in a craniocaudal direction, with the intact anterior cortex serving as a hinge. The exposed posterior cancellous bone surface with its intact anterior periosteal vascular supply provides an ideal osseous bed for new bone formation and autograft/allograft integration, which are required for a successful posterolateral spinal fusion. In case of a single-level fusion, the posterior-superior aspect of the inferior TP and the posterior-inferior aspect of the superior TP are decorticated, so as to provide the maximum bridging surface. When multiple levels are included in the fusion, either the superior or the inferior aspect of the posterior cortex of the TPs is decorticated by the surgeon, so as to minimize the risk of comminution and subsequent anterior devascularization during the unfolding maneuver. Alternatively, a rongeur can also be used to decorticate the dorsal aspect of the transverse process (Figure 2).

If lumbosacral posterior fusion is performed, besides the TP decortication and unfolding of the fifth lumbar vertebra, a “trap door” osteotomy is utilized in order to enhance the vascularized bone interface. For this osteotomy, one horizontal and two vertical cuts of about 1 cm are made to excise a bony fragment in each sacral ala. The bony fragment is not completely detached, preserving its ventral periosteal attachment and vascular supply, but is yet movable enough to be transposed and flipped proximally upon the exposed TP of the fifth lumbar vertebra. By using this technique, the bone vascularity is partially preserved, and the possibility of a successful lumbosacral fusion is increased, although caution is needed since L5 root injury can occur [17].

**Figure 3:**
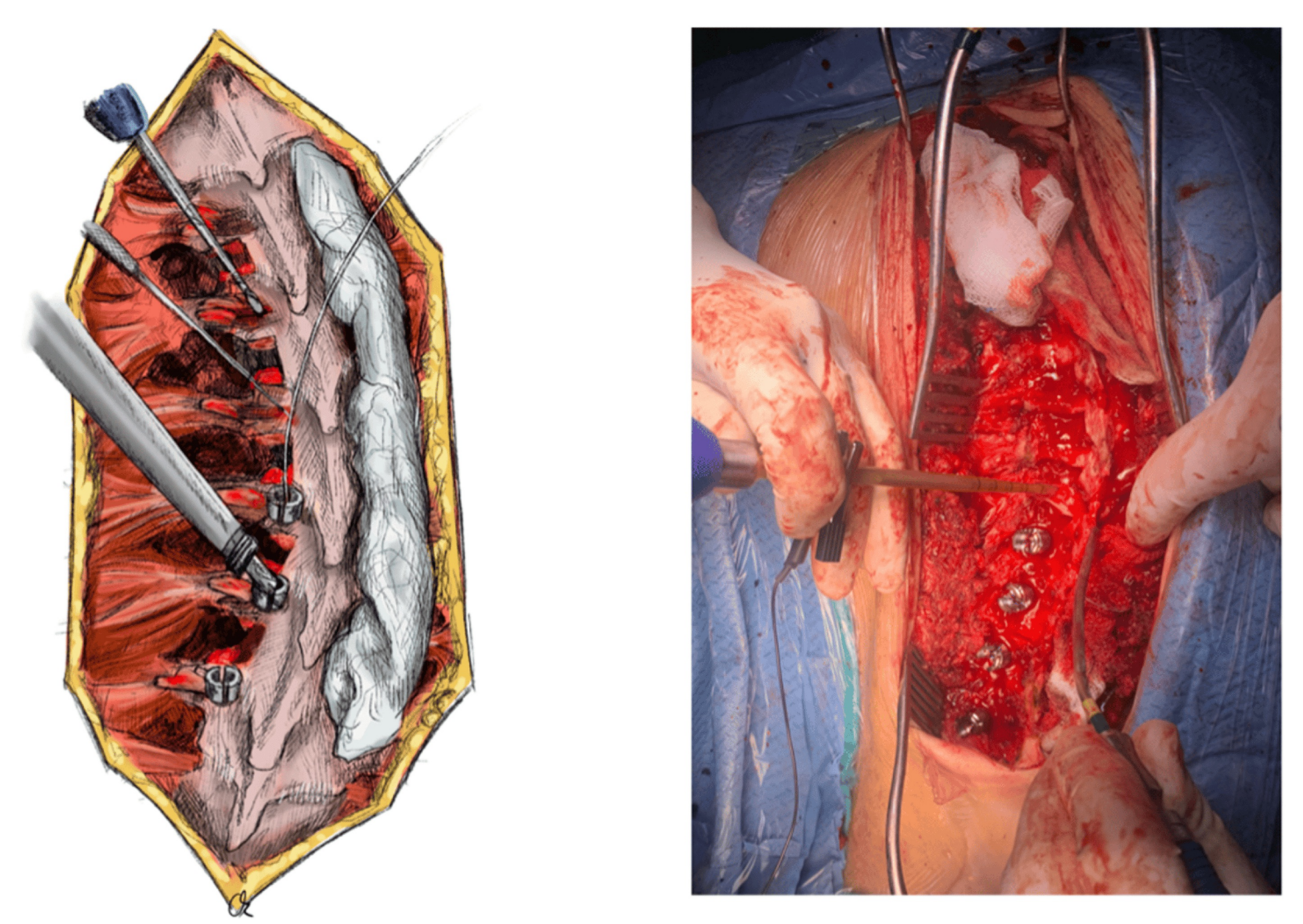
Pedicle screw placement through the decorticated pars and transverse process intersection A blunt straight probe is used to prepare each pedicle pathway, while a ball tip feeler is then used to confirm the integrity of the pedicular canal walls, as shown in the illustration (left) from cranial to caudal direction. At this point, intramedullary blood can be drawn from the created pedicular cavity of the vertebral body using a syringe serving as a liquid component rich in multipotent cells, which will be mixed with the allograft. Afterward, pedicular screws are inserted in each pedicle of the selected spinal levels, and fluoroscopy imaging is used to verify their correct placement. The whole process is performed under neurophysiological monitoring with electrodes, as depicted in the intraoperative picture (right). Illustration by Kimberly Ohm

After pedicle preparation for screw placement, intramedullary blood (13-15 mL) is drawn from the created pedicular cavity of the vertebral body using a syringe. The multipotential stromal cells contained in the medulla are reported to have a very high osseo-genic potential; therefore, intramedullary blood is preferred over peripheral blood as the liquid component to be mixed with the graft material [18]. Afterward, pedicular screws are inserted in each pedicle of the selected spinal levels, and fluoroscopy imaging is used to verify their correct placement. The whole process is performed under neurophysiological monitoring with electrodes (Figure 3).

**Figure 4:**
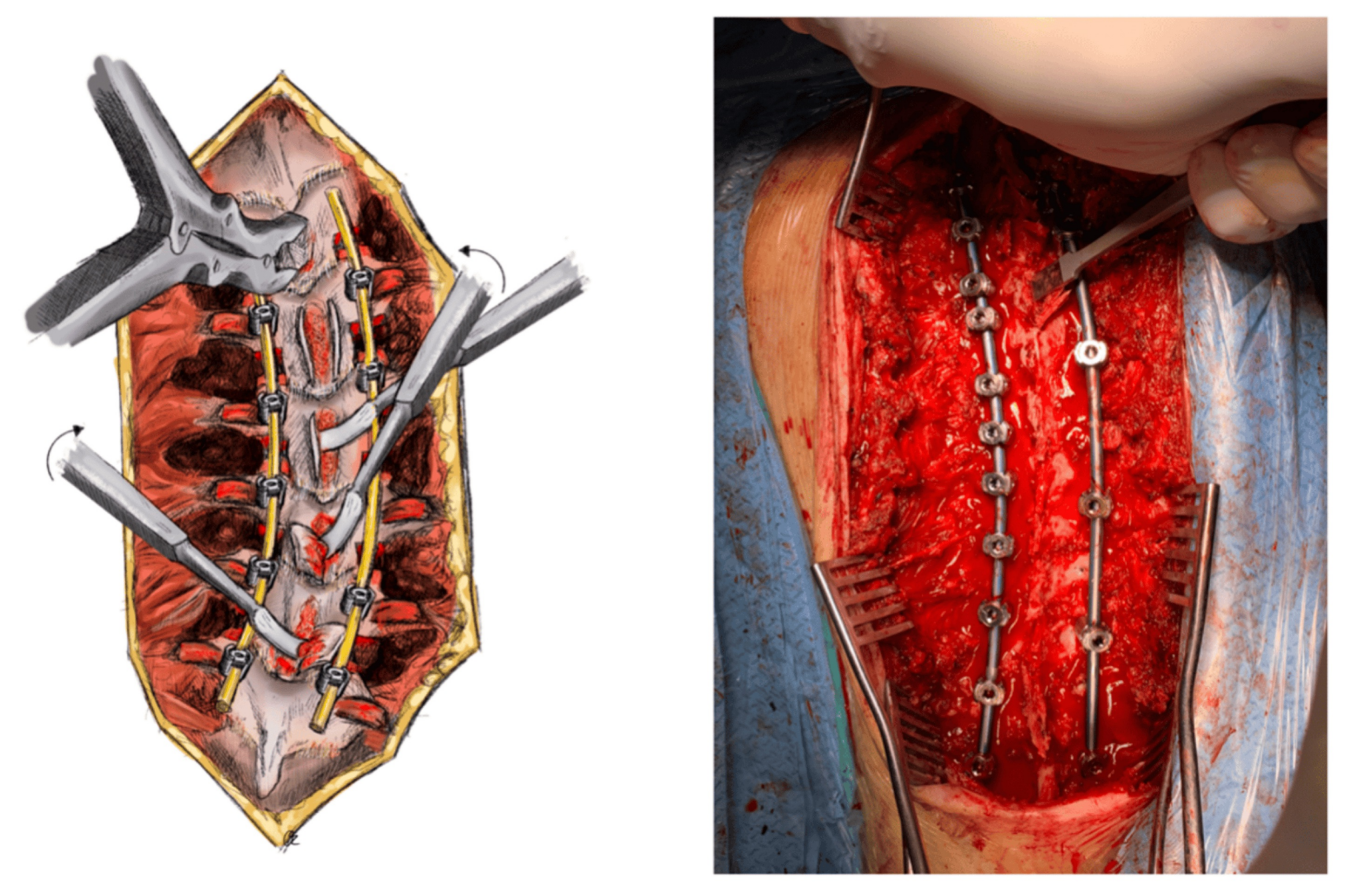
Spinous process hemi-resection and vertebral laminae decortication The supraspinous and interspinous ligaments are removed from their respective attachments upon the osseous part of the spinous processes. The spinous processes are then excised using a rongeur and are harvested as autologous bone graft, as shown in the illustration (left). The posterior cortex of all laminae is being raised and unfolded from a medial to lateral direction with the use of a curved osteotome, starting cranially and moving caudally, with special caution not to disrupt the periosteal attachment on the lateral edges, as depicted in the illustration (left) and the intraoperative picture (right). Illustration by Kimberly Ohm

After successful screw placement, the supraspinous and interspinous ligaments are removed from their respective attachments upon the osseous part of the spinous processes. The spinous processes are then excised using a rongeur and are harvested as autologous bone graft to be used later (Figure 4).

In case of idiopathic scoliosis, the coronal and sagittal deformity reduction with the use of pre-contoured rods (temporary or definite) followed by vertebral de-rotation maneuvers and additional corrective distraction or compression are performed, depending on the specific characteristics of the existing deformity being addressed [19].

After sufficient posterior soft tissue release and the completion of all correction maneuvers, the laminar decortication comes next. This part is usually reserved for the end because of the high risk for intraoperative bleeding [7]. The posterior cortex of all laminae is being raised and unfolded from a medial to lateral direction with the use of a curved osteotome, starting cranially and moving caudally, with special caution not to disrupt the periosteal attachment on the lateral edges. By doing so, this delamination technique is thought to preserve some of the laminar blood supply, therefore offering a better chance for new bone formation and eventually a solid posterior midline fusion (Figure 4).

**Figure 5:**
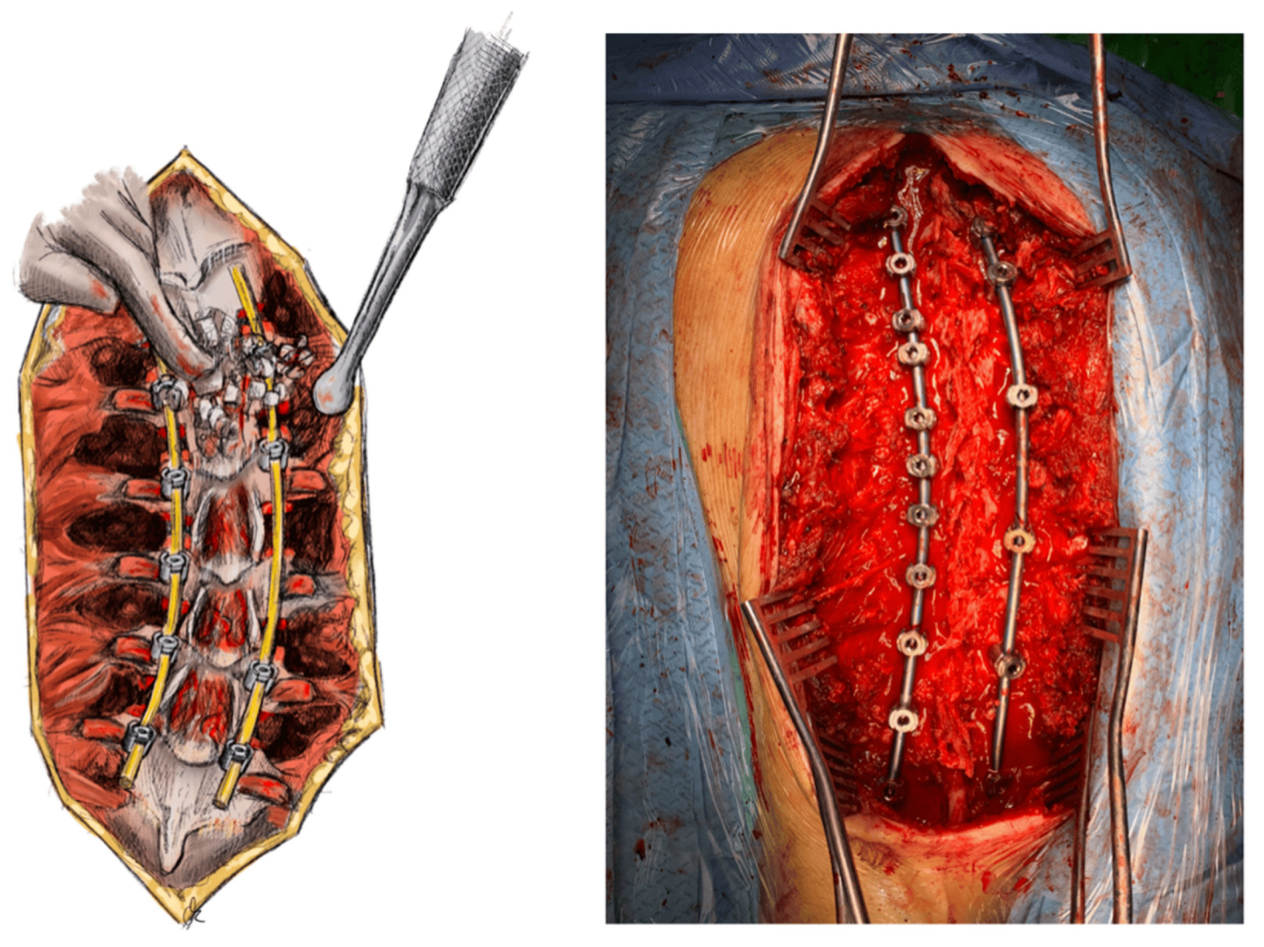
Graft material placement on the decorticated surface (delaminated midline surgical bed and posterolateral gutters) The homogenized graft material is placed between the facet osteotomies and onto the osseous surface of the unfolded transverse processes, in the posterolateral gutters right beneath the paraspinal muscles (posterolateral fusion), but also in the midline onto the delaminated osseous bed and interlaminar segments (posterior midline fusion), as demonstrated in the illustration (left). A completely decorticated cancellous bed is ideal to receive and integrate the autologous bone graft, as shown in the intraoperative picture (right). An additional layer of bioactive glass allograft impregnated with the pre-collected intramedullary blood may also be placed above the stuffed autologous graft, in order to minimize autograft displacement. Illustration by Kimberly Ohm

Finally, the bone chips that were harvested from the facet joint osteotomies, spinous processes, and interlaminar osteotomies are ripped of any remaining soft tissues and are homogenized with the use of a grinder. After thorough irrigation of the surgical site, the homogenized autograft is then stuffed between the facet osteotomies and onto the osseous surface of the unfolded transverse processes, in the posterolateral gutters right beneath the paraspinal muscles (posterolateral fusion), but also in the midline onto the delaminated osseous bed and interlaminar segments (posterior midline fusion). One layer of bioactive glass allograft impregnated with the pre-collected intramedullary blood is then placed above the stuffed autologous graft in the posterolateral gutters and the midline along the entire site between the rods, serving as a grout, in order to minimize autograft displacement (Figure 5) [20]. After placing two negative pressure drains underneath the incised fascia, the wound is closed.

## Discussion

Bone grafts in patients undergoing correction spinal surgery for scoliosis were first used in the late 1950s, when iliac crest bone graft (ICBG) was used to increase the fusion rate. The non-union rate in those patients in whom spinal fusion was initially performed without instrumentation was reported to be 28%, while this rate decreased to 18.1% with the introduction of Harrington instrumentation and further dropped to 2.7% when autogenous local bone grafting with decortication of the posterior elements was added. Local autogenous bone graft is easily accessible and costless, and can be safely used to achieve solid arthrodesis [21]. However, the question regarding the optimal type of bone graft remains still partly unanswered [22]. Based on our presented technique, autologous bone graft of homogenized laminectomy bone is placed as the first layer onto the decorticated osseous surfaces and covered with bioactive glass allograft impregnated with the pre-collected intramedullary blood from the pedicle screw pathways. Bioactive glass is mixed with bone marrow aspirate, which provides both precursor osteoblast cells and bone morphogenetic proteins, maximizing the graft’s potential for fusion [23,24].

Although the majority of published literature on this topic nowadays recommends autologous bone grafts as the ideal grafting material in spinal fusion, there has been much debate regarding the preferred type of bone graft. Many surgeons presume the iliac crest bone graft as the “gold standard” due to its well-established osteogenic, osteoinductive, and osteoconductive properties, providing an excellent fusion rate. However, due to the significant donor site morbidity associated with the iliac crest bone graft, some other surgeons prefer using autologous bone chips harvested from laminectomy and decortication sites. These autologous grinded bone chips also offer a high osteogenic potential and provide an osteoconductive scaffold with excellent osteoinductive properties [23].

Regarding autologous bone chips harvested from laminectomy and decortication sites, although previous studies in the past have reported that the fusion rate was unacceptable, the development and improvement in the technique of local bone preparation have led to an increase in the fusion rates [10]. In a recent study of 218 patients with adolescent idiopathic scoliosis (AIS) who underwent scoliosis correction surgery, the authors reported optimal results (>99%) in terms of spinal fusion with the sole use of local bone graft harvested during posterior element decortication, without any synthetic adjuvants [21]. According to several other studies, local bone grafts result in a fusion rate of 95%-99% in patients undergoing posterior spinal instrumentation for AIS [10,15,21,22]. In a large meta-analysis involving AIS patients, the authors concluded that iliac crest bone graft is associated with a higher rate of postoperative morbidity, while it confers no advantage over the other graft options in achieving fusion, therefore encouraging the use of non-iliac grafts and bone substitutes [10]. However, iliac crest bone autograft remains the “gold standard” for cases that require revision due to pseudoarthrosis or for comorbid patients with poor bone quality due to infection, neoplasms, or multi-segment involvement [25]. The same can be claimed for severe kyphotic deformity (e.g., Scheuermann’s kyphosis), in which Ponte osteotomies are not enough to achieve adequate sagittal plane correction, and therefore, pedicle subtraction or closing-wedge osteotomies in combination with hybrid instrumentation and iliac graft are needed to sufficiently correct the deformity and provide optimal spinal fusion rates [26].

There has been a great interest over the past decades in bone graft substitute options, with almost a third of the published literature focusing on bone morphogenic proteins (BMPs), while ceramics is also another area of extensive research [11,12]. Based on the current literature, BMPs are associated with the highest radiographic fusion rate in pediatric spinal deformity correction surgeries, followed by allograft and demineralized bone matrix [11,13]. Kadam et al. evaluated the application of beta-tricalcium phosphate (β-TCP) in scoliosis surgeries in adolescents, with a minimum follow-up of 20 months, and concluded that the use of β-TCP appeared to be a useful alternative to allograft in the AIS posterior instrumentation, in which large quantities of bone are required [11]. Novel synthetic bone substitutes have reliable osteoconductive potential and are widely used in spinal surgery, since there is no limitation in volume availability, and they are also easy to use. However, these synthetic allografts lack osteogenic and osteoinductive properties; hence, they are mainly used in combination with autografts as bone graft extenders. Bioactive glass is a novel type of ceramic material that offers excellent results when combined with autologous graft for spinal fusion; however, data regarding its efficacy as a standalone bone graft substitute are very limited and not promising [27].

In our institutions, we prefer to embed the bioactive glass graft in blood drawn from the pedicular cavity, as stem cells found in the bone marrow have the potential to self-renew, undertake clonal expansion, and differentiate into different musculoskeletal tissues [28]. Chaput et al. studied the vertebral fusion effects of mesenchymal stromal cells (MSCs) in 24 patients undergoing anterior cervical discectomy and fusion (ACDF) with the use of autologous concentrated bone marrow aspirate and allograft bone matrix. The reported one-year successful fusion rate was 86.7%, strongly correlating with a lower frequency of colony-forming unit fibroblasts in the bone marrow aspirate. The authors suggested that osteogenesis by human bone marrow is mostly dependent on the homeostatic ratio of MSCs to other cellular bone marrow components, whereas not so much on the absolute number of osteogenic MSCs, and they also concluded that a lower ratio of MSCs to other cellular components in marrow could predict effective osteogenesis during ACDF [29]. In their prospective case-control study comparing lumbar spine fusion rates between iliac autograft and a type 1 collagen/hydroxyapatite matrix soaked in bone marrow aspirate, Neen et al. found equivalent radiologic posterolateral lumbar fusion rates and clinical outcomes between the two groups. However, the radiologic fusion rates were lower regarding lumbar interbody fusions in the study group, despite the markedly reduced postoperative complications, mainly associated with the donor site morbidity in the control group [30]. Furthermore, studying the beneficial effects of additionally applying BMP-2 on the grafting material during adult spine deformity correction surgery, Onafowokan et al. demonstrated no superior outcomes of BNP-2 compared to a less costly biologic alternative (bone marrow aspirate + cancellous bone chips + i-Factor) in their large retrospective cohort of 512 patients [31]. Finally, although the strength and quality of current evidence is not yet solid and conclusive in favor of the standard use of bone aspirate in posterior spinal fusion surgery [32,33], the optimization of its adjuvant use in facilitating a more effective spinal arthrodesis could lie in the future exploration of cell populations and specific cell characterization, in order to determine the optimum cell for repair and/or regeneration of bone [28].

Thorough decortication and bone grafting are traditionally considered of utmost importance in spinal deformity correction surgeries, since a long-lasting spinal arthrodesis is the ultimate target of posterior instrumentation correction surgery. Bone graft integration is associated with certain local and systemic factors. The immediate contact of the bone graft with the exposed decorticated osseous surface allows a greater abundance of osteogenic factors in the interface between the recipient bed and the graft, inducing recruitment of osteogenic cells and neoangiogenesis [14,34]. Decortication enhances local metabolism in the contact area between bone graft and recipient bed by increasing the vascular supply to this region, accelerating bone graft integration with the recipient bed, and inducing greater new bone formation [14,34,35]. According to studies using markers of vascular neoformation, the fusion mass receives its primary blood supply from the decorticated transverse processes, while new bone formation is directly related to the decortication of the posterior bony elements [14,35]. However, many surgeons performing minimally invasive spinal surgeries (MIS) support that MIS without decortication/bone grafting is effective in achieving radiographic deformity correction, while reducing complications associated with traditional open surgery. However, the superiority of MIS over open surgical procedures with decortication and bone grafting procedures in terms of long-term fusion has not been evaluated yet in large, high-quality comparative studies [2,8].

## Conclusions

Scoliotic deformity correction surgeries are demanding procedures, while their success relies on robust long-term arthrodesis. However, the non-union rate, despite the recent advances in implant development and surgical techniques, is still high. In line with this, decortication and bone grafting can enhance the union rate, improving the results of these procedures, while advances in bone grafting technology have led to the development of new materials, such as novel ceramics, that can further decrease the revision rate in these surgeries. This technical note, presenting a reliable surgical technique for decortication and bone grafting that can be repeated in the same stepwise fashion, can be valuable for spinal surgeons. Critical steps include obliteration of the facet joints and decortication of the surfaces of the pars interarticularis, lamina, lateral aspects of the articulating facets, and transverse processes, as well as removal of all the excessive surrounding soft tissues. For bone grafting, the spinous processes of the vertebrae are hemi-sectioned at the base, divided in half on the sagittal plane, and serve as bone grafts. Further high-quality studies in large populations are needed to validate the results of this technique in terms of the long-term union rate in spinal deformity correction surgeries.
